# Retained in HIV Care But Not on Antiretroviral Treatment: A Qualitative Patient-Provider Dyadic Study

**DOI:** 10.1371/journal.pmed.1001863

**Published:** 2015-08-11

**Authors:** Katerina A. Christopoulos, Susan Olender, Andrea M. Lopez, Helen-Maria Lekas, Jessica Jaiswal, Will Mellman, Elvin Geng, Kimberly A. Koester

**Affiliations:** 1 HIV/AIDS Division, San Francisco General Hospital, University of California San Francisco, San Francisco, California, United States of America; 2 Columbia University Comprehensive HIV Program, Columbia University Medical Center, New York, New York, United States of America; 3 Division of Sociomedical Sciences, Columbia University Mailman School of Public Health, New York, New York, United States of America; 4 Center of AIDS Prevention Studies, University of California San Francisco, San Francisco, California, United States of America; Massachusetts General Hospital, UNITED STATES

## Abstract

**Background:**

Patients retained in HIV care but not on antiretroviral therapy (ART) represent an important part of the HIV care cascade in the United States. Even in an era of more tolerable and efficacious ART, decision making in regards to ART offer and uptake remains complex and calls for exploration of both patient and provider perspectives. We sought to understand reasons for lack of ART usage in patients meeting the Health Resources Services Administration definition of retention as well as what motivated HIV primary care appointment attendance in the absence of ART.

**Methods and Findings:**

We conducted a qualitative study consisting of 70 in-depth interviews with ART-naïve and ART-experienced patients off ART and their primary care providers in two urban safety-net HIV clinics in San Francisco and New York. Twenty patients and their providers were interviewed separately at baseline, and 15 dyads were interviewed again after at least 3 mo and another clinic visit in order to understand any ART use in the interim. We applied dyadic analysis to our data. Nearly all patients were willing to consider ART, and 40% of the sample went on ART, citing education on newer antiretroviral drugs, acceptance of HIV diagnosis, social support, and increased confidence in their ability to adhere as facilitators. However, the strength of the provider recommendation of ART played an important role. Many patients had internalized messages from providers that their health was too good to warrant ART. In addition, providers, while demonstrating patient-centered care through sensitivity to patients experiencing psychosocial instability, frequently muted the offer of ART, at times unintentionally. In the absence of ART, lab monitoring, provider relationships, access to social services, opiate pain medications, and acute symptoms motivated care. The main limitations of this study were that treatment as prevention was not explored in depth and that participants were recruited from academic HIV clinics in the US, making the findings most generalizable to this setting.

**Conclusions:**

Provider communication with regard to ART is a key focus for further exploration and intervention in order to increase ART uptake for those retained in HIV care.

## Abstract 2

In a qualitative study, Katerina Christopoulos examines the reasons for lack of ART usage in patients retained in HIV care.

## Editors' Summary

**Background:**

AIDS has killed about 39 million people since the first recorded case of the disease in 1981, and about 35 million people are currently infected with HIV, the virus that causes AIDS. HIV, which is usually transmitted through unprotected sex with an infected partner, destroys CD4 lymphocytes and other immune system cells, leaving infected individuals susceptible to other serious infections and to unusual cancers. Early in the epidemic, most HIV-positive individuals died from an AIDS-defining illness such as recurrent pneumonia, severe fungal infection, or Kaposi’s sarcoma (a type of cancer). Nowadays, although there is still no cure for AIDS, treatment with antiretroviral drugs (antiretroviral therapy or ART) can hold HIV in check, and, at least in affluent countries, HIV-positive individuals who are on ART now have a near-normal life expectancy.

**Why Was This Study Done?:**

HIV-positive individuals originally only started ART when their CD4 cell count fell below 200 cells/mm^3^ blood. As ART became more tolerable and more efficacious and as the harmful effects of viremia (HIV in the blood) became clear, the treatment threshold shifted upwards. Nowadays, the US ART guidelines, for example, recommend treatment for all HIV-infected individuals irrespective of cell count and, because ART reduces the risk of an HIV-infected individual transmitting the virus to an uninfected sexual partner, also endorse offering ART for the prevention of HIV transmission. However, 40,000 HIV-positive individuals in the US receive regular HIV care such as virus monitoring but are not prescribed ART, and 5% of individuals starting ART discontinue treatment. It is important to understand why HIV-positive individuals do not use ART, but decision making in relation to ART is complex, involving both the HIV-positive individual and their HIV care provider. HIV-positive individuals may refuse ART if they think their care provider does not believe they really need treatment, for example, and providers may not offer ART to someone they think will not adhere to treatment. Here, the researchers undertake a qualitative patient-provider dyadic study (an analysis of contrasts and overlaps between pairs of narratives) to investigate why some HIV-positive individuals are retained in HIV care but are not on ART and what motivates these individuals to attend HIV care clinics.

**What Did the Researchers Do and Find?:**

The researchers interviewed 20 HIV-positive patients (mainly men) attending two urban US HIV clinics who were either ART-naive or ART-experienced but off ART and their HIV care providers; 15 patient-provider dyads were re-interviewed after at least 3 months and another clinic visit. Patients were asked about their attitudes to ART, their ART usage, and their relationship with their provider. Providers were asked about their ART philosophy, how they discussed ART with their patients, and about their relationship with their paired patient. Nearly all the patients were willing to consider ART and 40% began ART during the study. Reasons given by patients for accepting ART included education about newer, more tolerable drugs but the strength of the provider recommendation of ART was particularly important in patient decisions to accept ART. Specifically, many patients had internalized messages from their providers that their health was too good to warrant ART. Providers for their part often muted the offer of ART to patients experiencing psychosocial problems who they believed would find it hard to adhere to treatment. Finally, patient interviews suggested that motivations for retention in HIV care in the absence of ART included access to viral monitoring and the patient-provider relationship.

**What Do These Findings Mean?:**

These findings may not apply to settings such as HIV clinics in sub-Saharan Africa where patients do not always see the same care provider or to women. Nevertheless, this study identifies the patient-provider encounter as a key factor in ART usage by HIV-positive individuals and highlights the importance of the discussion about ART between patient and provider in the patient’s decision to accept an offer of ART. Given these findings, the researchers make several recommendations about the role of the provider in ART initiation. For example, they suggest that provider assessment of how patients perceive their health status and the implications of ART initiation should be formalized as part of ART counseling. Attention to this and other aspects of the patient-provider encounter, they suggest, may help to improve ART usage among HIV-positive individuals and thereby increase both the individual and public-health benefits of ART.

**Additional Information:**

This list of resources contains links that can be accessed when viewing the PDF on a device or via the online version of the article at http://dx.doi.org/10.1371/journal.pmed.1001863.

- Information is available from the US National Institute of Allergy and infectious diseases on all aspects of HIV infection and AIDS, including the treatment of HIV/AIDS
- NAM/aidsmap provides basic information about HIV/AIDS, summaries of recent research findings on HIV care and treatment, and personal stories about living with HIV/AIDS
- Information is available from Avert, an international AIDS charity on many aspects of HIV/AIDS, including information on HIV/AIDS treatment and care and on treatment as prevention; Avert also provides personal stories about living with HIV/AIDS
- The World Health Organization provides information on all aspects of HIV/AIDS (in several languages), including its guidelines on the use of ART for treating and preventing HIV infection
- The *2014 UNAIDS World AIDS Day Report* provides up-to-date information about the AIDS epidemic and efforts to halt it

## Introduction

Maintaining HIV-infected individuals in consistent care is a priority in improving the lives and health outcomes of individuals living with HIV in the US [1]. The HIV care cascade demonstrates the importance of being linked to HIV care and attending regular visits in achieving the goal of virologic suppression [2]. Appointment attendance is necessary for antiretroviral therapy (ART) uptake; however, it is not sufficient. Patients who are well retained in care but not prescribed ART are a population of interest and according to recent estimates account for approximately 40,000 individuals [3]. A sobering finding from the Medical Monitoring Project (MMP) is that one in 20 individuals who ever start ART in the US discontinue ART [4]. Previous research has shown that barriers to ART initiation or reinitiation include a fear of side effects, unstable psychosocial situations, substance abuse, and conspiracy beliefs [5–7]. In addition, the patient–provider relationship affects ART use; a good relationship may promote ART uptake, while distrust can undermine it. Moreover, providers may withhold the offer of ART to patients they do not believe will adhere to medication [8,9].

In recent years, ART has become more tolerable and efficacious, and concerns about drug resistance have eased, prompting a series of changes in the Department of Health and Human Services (DHHS) guidelines. In October 2011, DHHS guidelines shifted the treatment threshold from a CD4 cell count below 350 cells/mm^3^ to a CD4 cell count below 500 cells/mm^3^, with the expert panel evenly split on recommending treatment above 500 CD4 cells/mm^3^. Growing evidence on the harmful effects of untreated viremia and the results of HIV Prevention Trials Network (HPTN) 052, which showed that ART decreased the risk of HIV transmission by 96% in serodiscordant couples, led to a revision of the guidelines in March 2012 that recommended treatment for all HIV-infected individuals, irrespective of CD4 cell count [10–12]. A subsequent update in February 2013 specifically endorsed the offer of ART for the prevention of HIV transmission and for the treatment of acutely infected individuals [13].

Despite data-driven guidelines in support of the individual and public health benefits of universal ART treatment, decision making in regards to the prescription and uptake of ART remains complex. ART offer and uptake involves both the HIV-infected individual and his or her primary care provider; thus, it is imperative to understand more about what transpires during the clinical encounters of those linked and retained in HIV care, but not on ART. The role of the provider in particular deserves exploration. A survey of ART-prescribing clinicians in the Bronx, New York, and Washington, D.C., in late 2010 found that only 55% recommended ART for patients with CD4 cell counts below 500 cells/mm^3^ [14]. The primary aim of this study was to explore barriers to ART uptake in a new era of ART from the perspectives of patients who are retained in clinic care and not currently on ART and their primary care providers. A second aim was to identify factors promoting regular primary care attendance for these patients in the absence of ART.

## Methods

### Study Design

We conducted in-depth semistructured interviews with dyads of HIV-infected patients and their primary care providers. We used the theoretical framework of dyadic analysis, which is phenomenological in that we sought to understand (1) the meaning patients and their providers assign to the lived experience of using and adhering to ART; (2) the dyadic experience of discussing and, often, negotiating ART; and (3) the decisions to encourage, prescribe, and initiate ART [15]. The theoretical orientation of dyadic analysis assesses contrasts and overlaps between pairs of narratives and has the potential to examine levels of awareness about what the other person thinks and feels [16], both important considerations given the study’s research questions. Because the offer and uptake of ART is subject to change over time, we conducted repeat interviews with patient/provider dyads. Repeat interviews were conducted after another clinical visit and at least 3 mo had passed from the initial set of interviews.

### Setting

The study was conducted at the HIV clinics of two academic medical centers in San Francisco and New York. Both clinics provide care to urban populations with public insurance but have different demographic and risk factor profiles, allowing for greater diversity in the study sample. At the San Francisco clinic, approximately 85% of the clinic population are men, two-thirds are men who have sex with men (MSM), and half are black or Latino. At the New York clinic, nearly 60% are men, 30% are MSM, and over 90% are black or Latino. In addition, it was likely that provider practice would vary by site. The San Francisco clinic has had a policy of a universal ART offer since 2010, while the New York clinic has more closely followed DHHS guidelines. Both clinics are actively involved in training HIV providers and have team approaches to medical care that include nurses, social workers, adherence counselors, and psychiatrists. This study was approved by the institutional review boards of the University of California San Francisco and Columbia University.

### Research Team

Two HIV physicians on the team (KAC and SO) helped recruit participants; graduate-level medical anthropologists and sociologists (KAK, AML, JJ, and WM) as well as an associate professor of public health (HML), all of whom were not known to patients or providers, conducted the interviews. The interviewers were all female, with one exception (WM), and most had extensive experience in conducting qualitative interviews (KAK, AML, and HML). Whenever possible, interviewers were assigned to either providers or patients in order to keep the interviewer’s approach uncontaminated from knowing the data from “the other side” and to facilitate the development of the follow-up interview guides.

### Participant Selection

Periodic reviews of the electronic medical record were conducted to identify patients without active ART prescriptions. We purposively sampled patients who (1) qualified for ART per the October 2011 DHHS guidelines, that is, they had a CD4 cell count less than 500 cells/mm^3^ at any time in the past year; (2) were not currently on ART, defined as not having taken ART for more than 2 wk in the past 3 mo; (3) were retained in clinic care as per the Health Resources Services Administration (HRSA) measure of two primary care visits at least 90 d apart in the past year [17]; (4) had been in clinic care for at least 6 mo, and; (5) were English-speaking. Providers were eligible if they were the primary care provider of an eligible patient, defined as having ART-prescribing privileges for that patient. Potentially eligible patients were identified through chart review. Eligibility was confirmed with the primary care provider, who was invited to participate in the study, extend the offer of participation to the patient, and secure approval for the study team to contact the patient. Patients and providers were advised that interview content would not be shared within the dyad. Providers could invite more than one patient to participate in the study.

The dyadic and longitudinal study design had implications for sampling participants until a point of saturation. We achieved saturation quickly among our provider participants but not with our patient participants. Our dyadic design meant that we needed to sample additional patients so that we could achieve saturation with both members of the dyad. We had permission from our institutional review board to interview up to ten patients per clinic site, and though we noted similar themes in the patient narratives after interviewing about 15 patients, we felt it was important to maximize our understanding as well as to verify our preliminary findings, especially given that several participants at each site were lost to follow-up after the initial interview. We ended up enrolling all 20 slots with an equal number of baseline patient interviews at each site.

### Data Collection

Study visits occurred during a mutually convenient time arranged during business hours. After the goals of the study were explained and participants provided informed written consent, face-to-face interviews took place in private rooms located in research space on a different floor than the clinic (San Francisco) or in the clinic itself (New York) and were audio recorded. In all cases, one interviewer conducted the interview privately with one participant. To accommodate schedules, providers were interviewed during administrative sessions, research time, or on lunch hours. Providers in San Francisco underwent phone interviews for follow-up, based on research showing no difference in data collected via phone or in person [18]. We used interview guides to direct the patient and provider interviews. For the patients, we covered the following topic areas: experiences with health care, HIV care, and HIV providers; knowledge of and attitudes towards ART; medication usage (ART and non-ART related); relationship with the provider, including a description of the last clinical encounter; and motivations for clinic attendance. Providers were asked to describe their ART philosophy and approach to discussing ART with patients and then to reflect on the particular patient. All interviews lasted 30–60 min (with follow-up interviews being shorter). Interviewers wrote field notes after each interview that provided a synopsis of the content of the interview and noted contextual factors not captured on the audio recording. Participants also completed a short demographic questionnaire, and the CD4 cell count and viral load closest to the initial interview was abstracted from the medical record. Patient participants received US$30 cash for the first interview and US$20 cash for the second interview, while providers received a US$20 Amazon gift card for the first interview and a US$10 Amazon gift card for the second interview. The study launched in San Francisco, and after three dyads were interviewed, a member of the San Francisco study team visited New York to conduct an on-site interviewer training. Regular conference calls were held during the first 6 mo of interviews in New York to provide interviewers with feedback and to ensure the interviews were yielding adequate data. The study team revised the baseline guide and developed the structure for the follow-up guide during these calls. A professional transcriptionist transcribed the recorded interviews verbatim. Study data were collected from June 2012–January 2014 in San Francisco and from November 2012–February 2014 in New York.

### Data Analysis

Three interdisciplinary members of the research team (KAC, KAK, and AML) read the data independently as they were transcribed and held regular analysis meetings, first monthly and then quarterly, during the duration of the study to discuss their respective interpretations of the data and collectively identify emerging themes. The dyad was the unit of analysis. We approached our dyads first by considering information in the patient interview and then turned to the content of the provider interview. We identified commonalities and points of divergence in the narratives, as well as convergence and discordance between how each member of the dyad perceived the other. A graduate-level anthropologist wrote a memo for each dyad that encompassed quotable quotes, observations from the group, and information from the field notes. Our analysis proceeded based on these memos. The three analysts then met weekly for 1 mo to review each dyad with regard to the reasons for lack of ART usage, attitudes towards and knowledge of HIV and ART, and the patient–provider relationship. The first author (KAC) sought informal feedback on the key findings from her clinician colleagues through internal presentation of study results. We used their input as a validity check of our interpretation of the provider findings. We had not built in a procedure to seek feedback from patient participants but will consider doing so in future studies.

## Results

### Participant Characteristics

All providers and patients approached agreed to be in the study, although we were unable to make contact with one potential patient participant. We recruited 20 dyads (ten at each site) as planned, which included 15 unique providers (eight in San Francisco and seven in New York). The median age of patient participants was 38 y (range of 25 to 60 y). Patient participants were mostly male (Table 1) and MSM and were 80% racial/ethnic minorities. There were high levels of self-reported drug use (40%) and prior incarceration (55%). The median time since HIV diagnosis was 8 y (range of 2–29 y). The median CD4 cell count in San Francisco was 402 (range of 204–604) and 361 in New York (range of 91–903). Six patient participants in San Francisco and four in New York were ART naïve (50% of patient sample). Of the 15 provider participants, eight were medical doctors (MDs), and seven were nurse practitioners (NPs) (Table 2). With regard to HIV primary care, three provider participants had been practicing for less than 5 y, five for 5–15 y, and seven for over 15 y.

**Table 1:** Patient characteristics (*n* = 20).

Category		Result
Age (median, range)		38 (25–60)
Years since HIV diagnosis (median, range)		8 (2–29)
Gender		
	Male	17 (85%)
	Female	3 (15%)
Race/ethnicity		
	White	4 (20%)
	African-American	10 (50%)
	Hispanic	5 (25%)
	Other	1 (5%)
HIV risk factor		
	Men who have sex with men (MSM)	10 (50%)
	Heterosexual sex	3 (15%)
	People who inject drugs (PWID)	2 (10%)
	PWID/MSM	2 (10%)
	Not sure/other	3 (15%)
Last CD4 count (median, range)		
	San Francisco	402 (204–604)
	New York	261 (91–903)
Housing stability		
	Own/rent	11 (55%)
	Live with family	2 (10%)
	Marginally housed	5 (25%)
	Homeless	2 (10%)
Prior psychiatric diagnosis		7 (35%)
Prior or current drug use		8 (40%)
Ever incarcerated		11 (55%)
ART naïve		10 (50%)

**Table 2:** Provider characteristics (*n* = 15).

Category		Result
Gender		
	Male	6 (40%)
	Female	9 (60%)
Degree		
	Medical doctor (MD)	8 (53%)
	Nurse practitioner (NP)	7 (47%)
Years from post-graduate training		
	<5 y	3 (20%)
	5–15 y	5 (33%)
	>15 y	7 (47%)
Years practicing HIV primary care		
	<5 y	3 (20%)
	5–15 y	5 (33%)
	>15 y	7 (47%)
Race/ethnicity		
	White	11 (73%)
	Nonwhite	4 (27%)
Patients seen per week		
	<25	10 (67%)
	25–50	4 (27%)
	>50	1 (7%)
Size of HIV primary care panel		
	<50	5 (33%)
	50–100	4 (27%)
	101–200	4 (27%)
	>200	2 (13%)

### Follow-up

We were unable to obtain follow-up for five patient participants. In New York, one participant was incarcerated, and repeated attempts to schedule another participant for follow-up were unsuccessful, essentially constituting a decline of the invitation for a second interview. In San Francisco, we could not reach one participant by phone, and his provider retired; another participant moved out of state; and a third participant (an elite controller with an undetectable viral load) felt an additional interview would not yield more information. Thus, we conducted 70 interviews in total (20 initial patient/provider interviews and 15 follow-up patient/provider interviews).

### Overall Concordance between Patient and Provider Narratives

Patient and provider explanations of why patients were not on ART were largely aligned. There were no cases in which the provider was unaware of or misinterpreted the patient’s reasons for not being on ART. Many patients did not think they needed ART or voiced a desire for more life stability prior to ART initiation. However, in many of these situations, the reason for not being on ART appeared to stem more from the provider than from the patient. Indeed, the extent to which the offer of ART was on the table in the patient–provider encounter ranged from a strong and consistent recommendation to an acknowledgment that ART was not necessarily the most important issue at hand. The reasons for not actively offering ART were varied. In some cases of higher CD4 cell counts and low viral loads, a few providers were not convinced of the individual health benefits of starting ART. Providers were also aware that patients had competing priorities. In addition, reasons patients gave for not being on ART were fears of the side effects and toxicities of ART and lack of acceptance and/or disclosure of HIV diagnosis and concerns about stigma. A few patients stated that they would never initiate ART, and in some of these cases, providers stopped raising ART as a possible treatment option.

### Provider Messaging and the Patient Response

While some providers endorsed and promoted a universal offer of ART regardless of CD4 cell count, others were less convinced that ART was indicated—at least urgently—in patients whose last CD4 cell count was over 500 or those with low viral loads. One provider viewed the DHHS guidelines as focused on decreasing HIV transmission at the population level, rather than on the individual patient.

I don’t think there’s a lot of scientific basis for that as a treatment recommendation. I think that is a public health recommendation. And I think that a physician walks a little bit of a tightrope because you have to view the patient as part of society, and so you understand they’re interacting with the rest of the world and in some cases spreading HIV all over the place. But they still have to be your first concern, so if you truly believe they can’t take ARVs [antiretroviral drugs] correctly, then you have to realize you could do them a tremendous disservice by prescribing them. And you also really need to look at the individual medical benefit to be gained. For some of the people who are kind of borderline for treatment guidelines but have very low viral loads, I’m less excited about getting on the bandwagon.

–Female MD with >15 y HIV primary care experience

The patient in this dyad had been on ART during pregnancy but was now off after a history of intermittent use. She was trying to regain custody of her child and in treatment for depression. The provider stated that the presence of these psychosocial issues led her to dissuade the patient from ART at this point, rather than simply not offer it.

Coupled with her past nonadherence and what I think are weak indications for medical treatment, I’m actually discouraging her from taking antiretrovirals. I think she’s got enough on her plate.

–Female MD with >15 y HIV primary care experience

The patient echoed that her provider had told her ART was not currently indicated.

She tells me that I don’t need it because my body’s handlin’ it…. When I see that my numbers are—like, my T cells are, like, under 200 or 250, then I’ll consider it. Or my viral load is over 100,000 or something like that, then I’ll consider it. But if it’s only 900 and 6,000, I don’t think those are numbers to consider starting it.

–27-y-old heterosexual African-American woman

The patient went on to say that in general, she was not opposed to ART.

I’m not against taking the medication. I’m more than willing to take it when the time is right for me. And when the time is right for my—and the doctor feels like the time is right for me to be put on it as well.

While this dyad represents a strongly articulated position on deferring ART, we found that a common theme for many patients was that they had internalized the message from the providers that their health was stable and that it was not yet time to start a lifelong medication regimen. A subset of these patients expressed a genuine willingness to initiate medications if their providers clearly stated that it was time to do so. For others, the willingness to initiate was dampened by what ART had symbolized for many years—a deterioration in health status.

One provider reflected on how the shift in guidelines confused the meaning of ART for patients and, in particular, for a patient of hers who had been living with HIV for over 20 y.

So they’ve been told for years, “You don’t need treatment. You don’t need to worry about that. Your CD4 count is high enough that you’re protected from opportunistic infections. We’ll keep monitoring, but you really don’t need to worry about treatment. It’s not time.” So to go from that—he was diagnosed in the ‘80s—and to have 20 years of people saying “you don’t need it” to the next day, “You know, we’ve had some new data come in, and we really think you do need it.” So those people are like, what? It’s a huge shift for them. Suddenly, their identity was completely changed. They’ve gone from, “Hey! I’m somebody whose immune system is so good that I don’t need treatment!” to “I need treatment. They tell me I need treatment. Wait a minute. But they were just telling me all these years I didn’t need treatment. So wait. I feel like the rug just got pulled out from under me, and I was feelin’ pretty solid on that rug.”

–Female NP with >15 y HIV primary care experience

Like the previous patient, this provider’s patient stated that he was willing to take ART when the time was right.

I got no problem with the meds. It’s just that I don’t feel that I’m ready. I don’t think my disease is at that point that I need to start takin’ ‘em. I think—‘cause I have a lot of friends on medication—that it make tremendous differences in their lives. I can see the life difference. It’s life changing—puttin’ on the weight and—just to get out the house—they didn’t look like they was at death’s door no more. Some of them went back to work. Yeah, I got no problem with the meds.

–44-y-old heterosexual African-American man

Importantly, both of these patients believed in the benefits of ART but did not see themselves as needing ART yet, a status their providers had also communicated to them. In fact, nearly all of the patients in the dataset were willing to consider ART. There were two “negative” cases of patients who stated they would never take ART because they had seen loved ones suffer and die in the early years of ART, specifically because of azidothymidine (AZT).

The people who were diagnosed when I was, they all jumped on the AZT bandwagon and were dead within a few months. I think that drug was designed to kill them…. I’m one of those conspiracy theorists. I know the government is designing something that’s speeding up the process. There’s no doubt in my mind.

–44-y-old white MSM

Because of this patient’s stance, his provider found that he did not bring up ART at every single encounter.

I try to bring it up very routinely. But I don’t talk about it all the time. Partly because I just see his belief system as so fixed. Although I do know the literature around—like tobacco smoking—and I’m sure it’s true with ART—that the more it’s brought up and talked about, the more likely somebody is to go on it…. But I don’t do that every visit, partially because I see him a lot for the pain prescriptions—he comes in monthly. But I would say every several months, every 3 months or so, we at least have a conversation around it.

–Male NP with >15 y primary care experience

### Life Stability—HIV Care Is More Than Just ART

As in the first dyad above, many providers acknowledged that there were complex psychosocial circumstances that often took precedence over HIV and ART. Patients and providers used the word “stability” or the phrase “more stable” when describing situations involving other medical comorbidities, homelessness, substance use, mental illness, and chronic pain and essentially indicated that ART would become more of a priority when these other issues had stabilized.

In my mind, he’s a case where, yes, by the numbers he should be on medicine. But he’s not, for reasons that to me make a lot of sense. He’s engaged in care because he really desperately needs emotional support, and I think that’s contributed to his life despite the fact that he hasn’t been on ART. ART is not the end-all be-all of caring about a human being who has HIV. In his case, I care about him and hope he will go on ART, but even if he didn’t, I still care about him. I still affirm him when he comes to the clinic. It just hasn’t been his priority or my priority in his care…. So I don’t feel in the least bit bad that he hasn’t started ART yet. I think he would have failed. I think it would have given him another reason not to like himself. His alcoholism is much more worrisome to me than his HIV, in terms of what is going to kill him. So my priorities have been to support his sobriety, support his psychiatric well-being.

–Female NP with >15 y HIV primary care experience

This provider presented a cogent reason for not pushing ART and focused on the stability of the patient’s overall health, not just his HIV health. Because of this holistic approach, the patient expressed his appreciation of his provider’s care.

I trust her with my life. If I was at the point where I was out and couldn’t make a decision, then if [she] said what to do, then that’s what to do. That’s how I feel.

–47-y-old white MSM

This patient did begin ART between the first and second interviews but relapsed with alcohol and was hospitalized for a suicide attempt, at which point he stopped ART. During the second interview, he stated that he needed to focus on his mental health and staying sober but would consider ART in the future.

An important nuance of the stabilization narratives was that patients also internalized and deployed the message that not taking ART consistently could result in drug resistance. Part of the shift towards readiness to start ART had to do with patients believing that they could take ART without missing doses. A younger patient with diabetes described this fear of “messing up.”

I can forget to give myself a shot of insulin and I’ll be okay. So this is more, oh, you can’t forget to take your medicine. If not, you’ll be screwed. So that idea freaks me out.

–28-y-old Hispanic MSM

Even outside of the context of life stabilization, providers described not wanting to drive patients away by focusing exclusively on ART in the clinical encounter.

Interviewer: You said that you’re not going to push them too hard. But what are your concerns about pushing them too hard? What would the consequences be?

Provider: I don’t want them to stop coming to see me. I at least want them to stay engaged with care so that we can keep monitoring their viral load and CD4 count and do risk-reduction counseling, because these guys are also having sex with other people. Even if they have low-level viremia, it’s still transmissible. And I do also talk to them about the benefits to their partners—by taking HAART [highly active antiretroviral therapy]—but if they’re still not willing to take it, I want to reinforce those things, do STD [sexually transmitted disease] screens, that kind of thing—harm-reduction stuff while they’re not on it. And I think, yeah, my goal is to get everyone on therapy, but there [are]a couple of people who just don’t want to be on it.

–Female MD with <5 y HIV primary care experience

### The Illness Experience: Suffering, Self-Worth, and Social Support

Many patients described complex narratives of psychological and physical suffering, low self-worth, and internalized stigma about HIV. Several patients had not disclosed their diagnosis to family members and feared negative responses; these patients had privacy concerns about storing ART in their homes. One patient who had previously been on ART but said that he discontinued it because of not having fully accepted his HIV diagnosis described how having the support of people at his job allowed him to restart ART.

I’ve talked to my boss’s wife and I’m really close to her, and she knows—I have to let her know everything—so she’s just like, oh my god, we’re here for you to give you the support you need. And since I received that, I was like, okay, I can do this. Go back to the doctor and take a more proactive approach now. I think because I felt like I had someone just there. It’s different when you don’t need—I’ve always felt like I’ve been doing this by myself. And it can be hard. You don’t feel like you have someone to talk to, just to get it out. It’s really hard. It’s a fear, the rejection, people rejecting you because of the stigma that comes behind having HIV, so you’re afraid of that, and I think I was really afraid of that.

–34-y-old African-American MSM

### Who Went on ART and Why?

Including the patient above, eight participants, including three of the ten ART-naïve patients, went on ART by the time of the second interview. For one ART-naïve patient who had been living with HIV for over 15 y, his turning point was joining a peer education program, after which he underwent training to become a health coach. This patient’s view of ART, which had been shaped predominantly by AZT, shifted as he learned that new medicines were less toxic. He also described overcoming his fear of not being able to take medication consistently and the importance of having a positive attitude.

I started to see that lifestyle-wise I could adapt to it. I can deal with it. Emotionally and physically, ‘cause of the way I’m active and stuff. I’m feelin’ courageous.

–37-y-old Hispanic MSM

In fact, this patient’s knowledge about ART increased so much through his job training that he became an ardent proponent of the potential public health role of ART.

When you find out, let’s get you on it right away. That’s our approach now, and that’s what we talk about at the seminars, so that—no. Get your client to get on those meds now. Not tomorrow, next week. As soon as possible. ‘Cause that’s gonna decrease the community viral load.

In contrast, the two other ART-naïve patients did not have reasons that were as well articulated for starting ART. These patients were younger and had been diagnosed with HIV in the past 3 y. One patient voiced empathy for his provider on the topic of ART.

Well, she sees me getting sicker and sicker, being more exhausted and looking tired as fuck—she’s so over it! I would be too. Take the damn meds!

–24-y-old white MSM

The diabetic patient who spoke about his fear of “messing up” appreciated that his provider (a female provider with 5–15 y of experience) did not push him on the subject of ART.

I do like the fact that almost a hand-holding, not forcing me to say—oh, no, you have to go on meds, now, it’s a life or death situation—I do like the fact that it’s a gradual process…. I got my last blood results and they were lower—I mean, upper 300s. So I’d been talking about it for a few years now with my doctor, so I just kind of gave into that. I didn’t want to start treatment later and then have things get extremely worse.

–28-y-old Hispanic MSM

The language of “giving in” was used by two other ART-experienced patients who restarted therapy, mostly because of symptoms of fatigue. For one patient, the ability to maintain ART in this context seemed more tenuous.

I guess I just relented. I relented, meaning I gave up and I finally said, all right. We’ll do it your way. And now I’m telling her: your way is not doing any benefit. I don’t see it…. I don’t see my health getting better. In fact, I see the opposite. I’m much more tired. I’m much more fatigued.

–45-y-old Hispanic MSM

Another ART-experienced patient mentioned in the first interview that he was aware that the “more sick” someone was, the more entitlements that would be available.

I sort of got to the point, too, like I was dealin’ with some problems, I’m like, look. Maybe I should not take the meds anymore, because I guess in this state—I don’t know why they consider you to have to be almost dying to be—to get help from the government, like housing assistance.

–31-y-old African-American MSM

At follow-up, he invoked fatigue as the reason he restarted therapy.

‘Cause even though you’re not taking medicine, your body’s still fighting, trying to fight it. But once you take the medicine and become undetectable, the body is at rest, sort of, because the medicine is whatever, it’s just keepin’ it—balance. But then when you’re not takin’ the medicine you’re sort of—it’s always a fight with the body, so the body’s tryin’ to fight it and do what it has to do. So I could see that my body was sort of tired, I guess.

However, his provider had a slightly different interpretation.

He wanted his T cell count to fall below a certain level so he would get housing. And that, as I said in the last interview, made me angry and resentful, and I think showed me that, again, he doesn’t have a complete understanding about what’s going on with his health…. He had a walk-in visit where he had respiratory complaints and had a chest X-ray that was consistent with pneumonia, which can be an AIDS-defining illness. And then he also had some thrush in his mouth, which also—he had some sore throats, suggestive of candida esophagitis, and together, those things got him the diagnosis of AIDS, which allowed him to get housing. So I don’t know necessarily whether his perspective really changed or whether we were just able to get him what he needed.

–Male MD with <5 y HIV primary care experience

This provider went on to acknowledge that the patient had other health goals he felt were secondary to getting back on ART but that he tried to meet the patient where he was in order to get him back on ART.

I do think a little bit with him is negotiating, and if my major priority is getting him to be on antiretrovirals, I’m willing to give him a few things in return. So I prescribed a topical acne treatment, which I probably wouldn’t have prescribed to other patients, and it’s not dangerous at all, but I don’t know whether it was really indicated, but I think that kind of exchange sometimes makes things work a little bit in our relationship.

The experience of this dyad illuminates not only the emotional weight but also the internal conflict providers feel when the patient and the provider have drastically different but well-reasoned goals relative to health. This patient had a strategy around ART in the context of his housing instability and limited resources. His provider disapproved of this strategy but was able to acknowledge the patient’s logic and to continue to support the patient in his efforts to engage in care despite the sense of discord it created for him.

### Reasons for Primary Care Visit Attendance in the Absence of ART

The second objective of our study was to understand what motivated clinic attendance in the absence of ART. The most common reason was to actively manage one’s health, particularly through lab monitoring.

What keeps you coming back is, you’re gonna need to come back because something might happen, you might catch a cold, you might get sick, you need to know where you are, basically, and you need to know where your T cells are and things like that, so I need to know what it was, how my body’s doin’, ‘cause every time that I come, every 2 or 3 months you take the bloodwork just to see where you are. You have to. You have to keep knowing where you’re at.

–31-y-old African-American MSM

Another common reason for attending primary care visits was that the patient felt a genuine connection with his or her provider. One patient compared seeing his provider to opiate replacement therapy.

It’s like—I come in, she’s my dose. Like somebody on methadone maintenance or something for heroin addiction, that’s my dose. I come to get my pill.

–51-y-old African-American former injection drug user (IDU)

Providers gave similar reasons for continued engagement.

In my mind, he got something out of the fact that he just had a place to get his numbers monitored, even though he didn’t do anything about them. And a place where he felt safe and not judged and not alienated by the fact that what he believed was very different from what I believed.

–Female NP with >15 y HIV primary care experience

Indeed, there was only one patient who voiced dissatisfaction with his provider. Additional reasons patients gave for coming to appointments were to access social services, to obtain opiate pain medication, and to address acute needs, such as STD symptoms.

## Discussion

In this qualitative study of HIV-infected patients well engaged in clinic care but not on ART and their primary care providers, we found several reasons for lack of ART usage. As in a 2006 study from Vancouver, medication factors and lack of support/stigma contributed to patient reasons for not taking ART [5]. However, our dyadic approach, in which both members of the dyad were equal “experts” on the research question, allowed us to see that many patients were not on ART because of messages they had internalized from providers over the course of their care, namely, that their health was too good to warrant ART at the time. This major finding aligns with recent data from the MMP, in which investigators found that the most common self-reported reason for ART discontinuation in a representative sample of patients (51%) was a doctor’s advice to delay or stop treatment [4]. Another message patients internalized was that to miss doses of ART would lead to insurmountable drug resistance, a situation which they feared. In addition, a dyadic look at the data revealed that for some providers and their patients, ART was not the most important priority in overall efforts to promote health and well-being; rather, providers prioritized psychosocial challenges and other comorbidities over ART. This consideration of the patient’s biopsychosocial context is a key feature of a patient-centered approach to providing medical care [19]. It is worth noting that in our small sample there did not appear to be a difference in willingness to prescribe ART by length of time in practice, i.e., that providers in practice longer deemphasized the offer of ART. This finding is concordant with the results of a clinician survey published by Kurth et al. in 2012 that found no significant differences in ART prescription practices by age, gender, or clinician type [14]. Finally, for some patients, it appeared that initiating or reinitiating ART had to do with a gradual journey in which lack of self-efficacy for ART, low self-worth, and psychological suffering were ameliorated to some extent by ART knowledge and increase in health literacy, acceptance of HIV diagnosis and oneself, and strong social support upon disclosure of HIV status.

Our dataset illustrated that movement toward taking ART was a fluid process in which attitudes towards ART and ART behavior did not necessarily remain static over the course of time. Interestingly, the reasons for not being on ART overlapped between treatment-naïve and treatment-experienced participants. Some of the treatment-experienced patients refused ART based on prior experience with side effects, missing doses, and pill taking as a reminder of having HIV. However, the treatment-naïve group raised similar concerns as fear of side effects and toxicities, fear of committing to a daily regimen, and fear of drug resistance. Both groups also mentioned coping with unstable life situations. The one area that appears to be unique to the treatment-naïve group is conspiracy beliefs, as it was invoked as a reason by the few individuals who stated they would never go on ART. We found that only two patients were not willing to consider ART but that many more were not ready. It was apparent that willingness and readiness constituted two separate states. Indeed, other investigators have proposed that not only are willingness and readiness distinct entities but that confidence in one’s ability to take ART is a third consideration [5]. As noted above, several patients initiated ART after overcoming fears about ability to take medication consistently. In addition, while many treatment guidelines recommend establishing patient readiness prior to ART initiation, a recent review found no uniformly agreed upon approach for how to assess readiness [20]. Potential approaches include assessments of knowledge, motivation, and where patients fall along the stages of change continuum. In addition, the findings of our study highlight that assessment of the belief systems that influence conceptions of readiness on both sides of the patient/provider dyad is critical.

In the absence of ART, reasons for primary care visit attendance were laboratory monitoring, strong connections to providers, social services offered by the clinic, receipt of opiate pain medication, and treatment of acute symptoms. Many patients in this study described feeling supported and cared for by their providers in important ways outside of what might be considered HIV care. This understanding of the whole person and the attempt to find common ground regarding management are key features of patient-centered medical care [21]. In an urban academic clinic similar to the ones in which this study was conducted, researchers found that patients kept more appointments if providers treated them with dignity and respect, listened carefully to them, explained in ways they could understand, and knew them as persons [22]. Thus, any efforts to promote patient retention in care while working towards ART initiation must continue to acknowledge the role of the patient–provider relationship.

Because this study was conceptualized in early 2011 before the results of the landmark HPTN 052 study were released, we focused on CD4 cell count in our eligibility criteria as opposed to using viral load, the presence/absence of a primary sexual partner, or sexual partner status. While we did ask patients if they had primary sexual partners, the partner narratives were not as well explored as aspects of HIV care, ART, and barriers to ART. In line with the spirit of qualitative inquiry, we did not want to “lead” patients into discussions of ART for prevention of transmission but, rather, wanted to see if patients raised this topic on their own, either because of discussions with their partners or with their providers. While some patients expressed awareness of ART as a tool for decreasing the risk of HIV transmission, most patients were not conversant on this topic, and the provider interviews revealed that it was not a standard part of the approach to ART counseling. In addition, as this paper was going to press, the interim results of the Strategic Timing of AntiRetroviral Treatment (START) study, a global, large-scale randomized trial comparing immediate ART in those with CD4 cell counts >500 cells/mm^3^ with deferring ART until CD4 cell counts fell below 350 cells/mm^3^, demonstrated a 53% reduction in the risk of serious AIDS/non-AIDS-related illness or death (http://www.niaid.nih.gov/news/newsreleases/2015/Pages/START.aspx). Research on how this evidence will affect both ART offer and uptake is warranted.

Another limitation of this study is that it is impossible to know the representativeness of our sample because it is impossible to know at the clinic level how many patients are not taking ART at a given point in time. While the electronic medical record contains ART prescriptions, the presence of a prescription does not mean that the patient is actually taking ART. Our patient sample contained few women, and additional exploration by gender could be instructive. Similarly, the providers with the most conservative ART-prescribing practices may not have referred patients to our study, as they may not have wanted to discuss their ART approach during a time of changing guidelines. Finally, our sample size was not large enough to definitively draw conclusions regarding differences in provider practices or patient knowledge, attitudes, and beliefs towards ART by clinic site. Given that the patient and provider samples were drawn from urban academic HIV clinics in the US, the results of this study are likely to be most generalizable to this kind of developed world setting. In sub-Saharan Africa, for example, it is unlikely that patients will see the same provider continuously or even at consecutive visits. Recent work in this setting has shown that ART refusal in treatment-eligible individuals occurs during linkage to care, rather than in the context of an established patient–provider relationship [23].

### Conclusions

Our study reveals that while the HIV care cascade depicts patients as the population of interest with regard to ART uptake, the critical site for exploration and intervention is the patient/provider encounter and specifically the discussion of ART. In our study, providers were practicing patient-centered care: they were sensitive to a patient’s life context, which often resulted in them (at times unintentionally) muting the offer of ART. Patients confirmed that providers had not strongly directed them to take ART but many indicated that such a clear recommendation would be necessary for ART initiation. As such, the findings of this study point to three potential recommendations with regard to the role of the provider in ART initiation. First, because some patients, especially those living with HIV for many years, strongly associated ART initiation with a serious decline in health status, provider assessment of how patients perceive their health status and the individual meaning of ART initiation should be formalized as part of ART counseling. As the medical anthropologist Arthur Kleinman has advised, this assessment can be framed as “What is at stake for the patient?” [24]. Applied to the issue of ART initiation, the approach could contain, among other things, understanding how the patient explains HIV and how HIV affects the patient’s body, the patient’s understanding of the role of ART, what taking ART would mean, and exploring fears about HIV and ART. The provider can then provide information about ART in the context of the patient’s concerns. A second point is that while the DHHS guidelines recommend identifying barriers to adherence prior to ART initiation, providers need specific strategies to address specific types of barriers. For example, as in this study, a provider may elicit that conspiracy beliefs are a barrier to ART initiation, but where does the conversation go from there? What methods might help the provider continue the conversation with the patient without the patient shutting down? In addition, it may be worth reassessing previously insurmountable barriers, e.g., the fear of drug resistance, given that the resistance profile of newer ART regimens may be more forgiving [25]. Third, instruction in techniques for providers to improve their communication in regards to the need and urgency for ART merits further attention. Several providers indicated to us that participating in this study gave them the occasion to reflect on their ART practices, and in so doing, they realized that they might not be offering ART as strongly as they believed it was indicated. In general, providers commented that having the opportunity to reflect formally on patients with different health goals could be beneficial, especially hearing what patients have to say about the patient/provider interaction. In the current era of multidisciplinary approaches to HIV care, case conferences that include the patient could potentially accomplish this goal. While outside the scope of this particular study, the role of other multidisciplinary team members in the decision to start ART should also be explored. Attention to these important issues may then allow providers to identify new ways to meaningfully engage with patients and continue the dialogue on ART.

## Supporting Information

S1 Table Consolidated criteria for reporting qualitative studies (COREQ): 32-item checklist. (DOCX)

## Abbreviations:

ART: antiretroviral therapy
ARV: antiretroviral drug
AZT: azidothymidine
COREQ: consolidated criteria for reporting qualitative studies
DHHS: Department of Health and Human Services
HAART: highly active antiretroviral therapy
HPTN: HIV Prevention Trials Network
HRSA: Health Resources Services Administration
IDU: injection drug user
MD: medical doctor
MMP: Medical Monitoring Project
MSM: men who have sex with men
NP: nurse practitioner
PWID: people who inject drugs
START: Strategic Timing of AntiRetroviral Treatment
STD: sexually transmitted disease

## References

[1] Office of National AIDS Policy, the White House. National HIV/AIDS Strategy for the United States. http://www.whitehouse.gov/sites/default/files/uploads/NHAS.pdf.

[2] Gardner EM, McLees MP, Steiner JF, Del Rio C, Burman WJ (2011) The spectrum of engagement in HIV care and its relevance to test-and-treat strategies for prevention of HIV infection. Clin Infect Dis 52: 793–800. 10.1093/cid/ciq243 21367734 PMC3106261

[3] Hall HI, Frazier EL, Rhodes P, Holtgrave DR, Furlow-Parmley C, et al (2013) Differences in human immunodeficiency virus care and treatment among subpopulations in the United States. JAMA internal medicine 173: 1337–1344. 10.1001/jamainternmed.2013.6841 23780395

[4] Hughes AJ, Mattson CL, Scheer S, Beer L, Skarbinski J (2014) Discontinuation of antiretroviral therapy among adults receiving HIV care in the United States. Journal of acquired immune deficiency syndromes 66: 80–89. 10.1097/QAI.0000000000000084 24326608 PMC5091800

[5] Alfonso V, Bermbach N, Geller J, Montaner JS (2006) Individual variability in barriers affecting people's decision to take HAART: a qualitative study identifying barriers to being on HAART. AIDS Patient Care STDS 20: 848–857. 17192150 10.1089/apc.2006.20.848

[6] Turner BJ, Fleishman JA, Wenger N, London AS, Burnam MA, et al (2001) Effects of drug abuse and mental disorders on use and type of antiretroviral therapy in HIV-infected persons. J Gen Intern Med 16: 625–633. 11556944 10.1046/j.1525-1497.2001.016009625.x PMC1495260

[7] Bogart LM, Wagner G, Galvan FH, Banks D (2010) Conspiracy beliefs about HIV are related to antiretroviral treatment nonadherence among african american men with HIV. J Acquir Immune Defic Syndr 53: 648–655. 10.1097/QAI.0b013e3181c57dbc 19952767 PMC2845717

[8] Bogart LM, Catz SL, Kelly JA, Benotsch EG (2001) Factors influencing physicians' judgments of adherence and treatment decisions for patients with HIV disease. Med Decis Making 21: 28–36. 11206944 10.1177/0272989X0102100104

[9] Loughlin A, Metsch L, Gardner L, Anderson-Mahoney P, Barrigan M, et al (2004) Provider barriers to prescribing HAART to medically-eligible HIV-infected drug users. AIDS Care 16: 485–500. 15203416 10.1080/09540120410001683411

[10] Mugavero MJ, Napravnik S, Cole SR, Eron JJ, Lau B, et al (2011) Viremia copy-years predicts mortality among treatment-naive HIV-infected patients initiating antiretroviral therapy. Clin Infect Dis 53: 927–935. 10.1093/cid/cir526 21890751 PMC3189165

[11] Cohen MS, Chen YQ, McCauley M, Gamble T, Hosseinipour MC, et al (2011) Prevention of HIV-1 infection with early antiretroviral therapy. N Engl J Med 365: 493–505. 10.1056/NEJMoa1105243 21767103 PMC3200068

[12] Reekie J, Gatell JM, Yust I, Bakowska E, Rakhmanova A, et al (2011) Fatal and nonfatal AIDS and non-AIDS events in HIV-1-positive individuals with high CD4 cell counts according to viral load strata. AIDS 25: 2259–2268. 10.1097/QAD.0b013e32834cdb4b 21918422

[13] Panel on Antiretroviral Guidelines for Adults and Adolescents. Guidelines for the use of antiretroviral agents in HIV-1-infected adults and adolescents Department of Health and Human Services http://aidsinfo.nih.gov/ContentFiles/AdultandAdolescentGL.pdf.

[14] Kurth AE, Mayer K, Beauchamp G, McKinstry L, Farrior J, et al (2012) Clinician practices and attitudes regarding early antiretroviral therapy in the United States. Journal of acquired immune deficiency syndromes 61: e65–69. 10.1097/QAI.0b013e31826a184c 23183150 PMC3957230

[15] Van Manen M (1990) Researching Lived Experience. Albany, NY: State University of New York Press.

[16] Eisikovits Z, Koren C (2010) Approaches to and outcomes of dyadic interview analysis. Qual Health Res 20: 1642–1655. 10.1177/1049732310376520 20663940

[17] Mugavero MJ, Davila JA, Nevin CR, Giordano TP (2010) From access to engagement: measuring retention in outpatient HIV clinical care. AIDS Patient Care STDS 24: 607–613. 10.1089/apc.2010.0086 20858055 PMC2965698

[18] Sturges JE, and Hanrahan K.J. Comparing telephone and face-to-face qualitative interviewing: a research note. Qualitative Research 4: 107–118.

[19] Lekas HM, Siegel K, Leider J (2012) Challenges facing providers caring for HIV/HCV-coinfected patients. Qual Health Res 22: 54–66. 10.1177/1049732311418248 21825278 PMC4323265

[20] Grimes RM, Grimes DE (2010) Readiness: the state of the science (or the lack thereof). Curr HIV/AIDS Rep 7: 245–252. 10.1007/s11904-010-0056-2 20714831 PMC2989238

[21] Mead N, Bower P (2000) Patient-centredness: a conceptual framework and review of the empirical literature. Soc Sci Med 51: 1087–1110. 11005395 10.1016/s0277-9536(00)00098-8

[22] Flickinger TE, Saha S, Moore RD, Beach MC (2013) Higher quality communication and relationships are associated with improved patient engagement in HIV care. Journal of acquired immune deficiency syndromes 63: 362–366. 10.1097/QAI.0b013e318295b86a 23591637 PMC3752691

[23] Katz IT, Dietrich J, Tshabalala G, Essien T, Rough K, et al (2015) Understanding treatment refusal among adults presenting for HIV-testing in Soweto, South Africa: a qualitative study. AIDS and behavior 19: 704–714. 10.1007/s10461-014-0920-y 25304330 PMC4393756

[24] Kleinman A, Benson P (2006) Anthropology in the clinic: the problem of cultural competency and how to fix it. PLoS Med 3: e294 17076546 10.1371/journal.pmed.0030294 PMC1621088

[25] Shuter J (2008) Forgiveness of non-adherence to HIV-1 antiretroviral therapy. J Antimicrob Chemother 61: 769–773. 10.1093/jac/dkn020 18256112

