# Supplementary material for: Retained in HIV Care But Not on Antiretroviral Treatment: A Qualitative Patient-Provider Dyadic Study
Source: PLoS Med. 2015 Aug 11;12(8):e1001863. doi: 10.1371/journal.pmed.1001863 (PMC4532493; doi:10.1371/journal.pmed.1001863)
Supplement: S1 Table — (DOCX) [file pmed.1001863.s001.docx]

**Supporting Information**

Consolidated criteria for reporting qualitative studies (COREQ): 32-item checklist

| **No** | **Item** | **Guide questions/description** |
| --- | --- | --- |
| **Domain 1: Research team and reflexivity** | | |
| Personal Characteristics | | |
| 1. | Interviewer/facilitator | KAK, AML, JJ, WM, HL |
| 2. | Credentials | KAK (MA), AML (MA), JJ (MPH), WM (MSW), HL (PhD) |
| 3. | Occupation | KAK (Research Specialist), AML (PhD Candidate in Anthropology), JJ (PhD Candidate in Sociology), WM (PhD Candidate in Sociology), HL (Associate Professor of Public Health) |
| 4. | Gender | All interviewers were female except for WM. |
| 5. | Experience and training | KAK, AML, and HL had extensive experience with conducting in-depth interviews for qualitative research studies; JJ and WM were doctoral students trained in qualitative interviewing. |
| Relationship with participants | | |
| 6. | Relationship established | None. |
| 7. | Participant knowledge of the interviewer | With provider participants, it is possible that some providers had met HML or KK through other research studies. |
| 8. | Interviewer characteristics | All interviewers are interested in qualitative research as it pertains to individuals living with HIV/AIDS |
| **Domain 2: Study design** | | |
| Theoretical framework | | |
| 9. | Methodological orientation and Theory | Dyadic analysis, phenomenology |
| Participant selection | | |
| 10. | Sampling | Purposive sampling |
| 11. | Method of approach | Providers were made aware of the study by research staff after electronic medical record query yielded potentially eligible patients. Once eligibility was confirmed with the provider and the provider agreed to be in the study, providers asked patients face to face or over the phone if they were interested in the study. Once patients agreed to be in the study, they were approached face to face or in person by a study researcher. |
| 12. | Sample size | 35 |
| 13. | Non-participation | We were unable to obtain follow up for 5 patient participants – one was incarcerated, one had a non-working phone number, one moved away, and two declined the offer of a follow-up interview. |
| Setting | | |
| 14. | Setting of data collection | Clinic/research exam rooms or offices. |
| 15. | Presence of non-participants | No |
| 16. | Description of sample | See Tables 1 and 2 |
| Data collection | | |
| 17. | Interview guide | An interview guide was drafted, piloted, and revised. |
| 18. | Repeat interviews | Thirty repeat interviews were conducted. |
| 19. | Audio/visual recording | Interviews were audio-recorded |
| 20. | Field notes | Yes |
| 21. | Duration | 30-60 minutes |
| 22. | Data saturation | Yes |
| 23. | Transcripts returned | No |
| **Domain 3: Analysis and findings** | | |
| Data analysis | | |
| 24. | Number of data coders | Three |
| 25. | Description of the coding tree | A coding tree was not developed. |
| 26. | Derivation of themes | Themes were derived from the data. |
| 27. | Software | No |
| 28. | Participant checking | Not with patient participants. KAC sought informal feedback from her clinician colleagues (some of whom were provider participants) through internal presentation of results, which constituted a validity check of our interpretation of provider findings. |
| Reporting | | |
| 29. | Quotations presented | Yes |
| 30. | Data and findings consistent | Quotations used to illustrate findings. |
| 31. | Clarity of major themes | Yes |
| 32. | Clarity of minor themes | Diverse cases noted and minor themes presented. |
